# On the Utility of Trisnitrate of Bismuth in the Diarrhea Accompanying Phthisis

**Published:** 1849-04

**Authors:** T. Thompson


*On the Utility of Trisnitrate of Bismuth in the Diarrhea accompanying Phthisis.* By T. THOMPSON, M.D., F.R.S., etc.—The author considers the trisnitrate of bismuth to surpass in efficacy and safety our most approved remedies for this complaint. He has taken every opportunity, during the last twelve months, of testing its powers, and has preserved notes of twenty-one of the cases in which it was administered. Of these, eighteen were phthisis in various stages of progress, and three bronchitis. In fifteen of the patients the diarrhea was entirely removed; in four transient benefit was experienced, and the remedy proved useless only in two instances. The dose administered was about five grains daily, usually combined with a little magnesia and gum arabic. Dr. Thompson has referred to various authors who have written respecting the properties of bismuth, without being able to collect from them any evidence of its powers in the phthisical variety of diarrhea, but he entertains a strong conviction of its peculiar appropriateness to this affection, and has obtained important confirmation of his experience in a recent communication from Dr. Lombard of Geneva.—*Dublin Med. Press.*—*N. Y. Jour. Med. and Col. Sci.*

